# Increased miR-124-3p alleviates type 2 inflammatory response in allergic rhinitis via IL-4Rα

**DOI:** 10.1007/s00011-022-01614-x

**Published:** 2022-08-03

**Authors:** Qian Liu, Yang Shen, Yifang Xiao, Hong Xiang, Ling Chu, Tiansheng Wang, Honghui Liu, Guolin Tan

**Affiliations:** 1 Department of Otorhinolaryngology—Head Neck Surgery, Third Xiangya Hospital, Central South University, Changsha, 410013 Hunan People’s Republic of China; 2 Department of Otorhinolaryngology—Head Neck Surgery, The Third Affiliated Hospital of Guangzhou Medical University, Guangzhou, 510510 Guangdong People’s Republic of China; 3 Center for Experimental Medicine, Third Xiangya Hospital, Central South University, Changsha, 410013 Hunan People’s Republic of China; 4 Department of Pathology, Third Xiangya Hospital, Central South University, Changsha, 410013 Hunan People’s Republic of China

**Keywords:** Allergic rhinitis, Inflammation, miR-124-3p, IL-4Rα, STAT6

## Abstract

**Background and objectives:**

miRNAs play a crucial role in regulating immune responses. However, the effect of miR-124-3p on type 2 inflammation in allergic rhinitis (AR) is unclear. We aimed to study the immune regulation of miR-124-3p in AR and the mechanisms involved.

**Methods:**

The direct interaction between miR-124-3p and IL-4Rα was confirmed through a dual-luciferase reporter assay. In vitro splenic lymphocytes from mice and peripheral blood mononuclear cells (PBMCs) from healthy individuals were cultured and treated with miR-124-3p mimic/inhibitor. Twenty-four female C57BL/C mice were divided into four groups: control, AR model, miR-124-3p agomir, and miR-124-3p antagomir groups (*n* = 6 per group). The allergic responses were evaluated based on the number of sneezing and nasal scratching, the serum HDM-specific IgE (sIgE) levels, and the degree of nasal mucosa eosinophil infiltration. The expression of IL-4Rα, p-STAT6, and type 2 inflammatory cytokines (IL-4, IL-5 and IL-13) in lymphocytes or nasal mucosa was determined by qPCR, western blotting, flow cytometry, immunohistochemistry and immunofluorescence.

**Results:**

miR-124-3p directly targets the 3'UTR of IL-4Rα. The miR-124-3p mimic lowered the IL-4Rα, p-STAT6, IL-4, IL-5, and IL-13 expression levels in both mouse splenic lymphocytes and human PBMCs in vitro, and the miR-124-3p inhibitor rescued these changes. Furthermore, the miR-124-3p agomir decreased the levels of IL-4Rα and IL-4 in nasal mucosa, Th2 differentiation in spleen, and allergic response in AR mice. Moreover, the miR-124-3p antagonist increased the IL-4Rα and IL-4 levels and further aggravated the allergic responses.

**Conclusions:**

miR-124-3p might attenuate type 2 inflammation in AR by regulating IL-4Rα signaling, and miR-124-3p may be a promising new target in AR treatment.

**Supplementary Information:**

The online version contains supplementary material available at 10.1007/s00011-022-01614-x.

## Introduction

Allergic rhinitis (AR) is an IgE mediated inflammatory disease with increasing prevalence [1, 2]. Patients with AR often experience symptoms of watery rhinorrhea, sneezing, itching and nasal congestion. Increasing evidence shows that type 2 inflammation is the main pathophysiology mechanism of AR [1, 3]. Although the ARIA (Allergic Rhinitis and its Impact on Asthma) guideline provides a global, evidence-based, pragmatic, stepwise approach to the treatment of AR [4], several patients are unsatisfied with the current treatments. Therefore, the identification of new therapeutic targets in AR is necessary.

MicroRNAs (miRNAs) are small noncoding RNAs of approximately 22 nucleotides that regulate most cellular processes via posttranscriptional control [5]. One miRNA can target multiple mRNAs, often within the same signaling pathway. Dysregulated miRNA expression may alter particular cellular functions and contribute to the development of various diseases [6]. In recent years, miRNAs have attracted extensive scientific attention due to their importance in the pathophysiology of allergic diseases. For example, the profiles of miRNA expression in allergic skin conditions and asthma were recently reported, and the results uncovered some allergic-related miRNA signatures, such as miR-21, miR-124, miR-151a, and miR-155 [7]. A subset of circulating miRNAs in plasma, miR-206, miR-338-3p, miR-329, and miR-26a, were found to be differentially expressed in patients with AR compared with healthy individuals or those with asthma [8]. Our previous study found that miR-124-3p was significantly decreased in the nasal mucosa of AR mice, and the expression was increased after anti-inflammatory treatment [9], demonstrating that miR-124-3p might be a target in AR. It has been reported that miR-124-3p alleviates nasal inflammation by inhibiting dipeptidyl peptidase-4 in allergic mice [10], but the effect of miR-124-3p on type 2 inflammation in AR is unclear.

In this study, we investigated the immune regulation of miR-124-3p in type 2 inflammation and the possible mechanism in AR.

## Materials and methods

### Mice

Six-to-eight-week-old wild-type female C57Bl/6J mice free of murine-specific pathogens were obtained from the Medical Experimental Animal Center of Central South University (Changsha, China). The mice were housed in the pathogen-free facility with a 12-h light/12-h dark cycle and free access to food and water. All procedures were approved by the Central South University Animal Ethics Committee.

### Isolation of human peripheral blood mononuclear cells (PBMCs)

This study was approved by the ethics committee of the Third Xiangya Hospital of Central South University (No: 2022-S132). All volunteers signed informed consent forms. Blood samples were collected from 12 healthy volunteers, including six men and six women without a diagnosis of AR and who had a negative skin prick test (SPT) or specific serum IgE (sIgE) measurement. Human PBMCs were prepared from venous blood using the Ficoll–Hypaque method (TBD, China). The isolated human PBMCs were used for in vitro culture.

### AR mouse model and treatment protocol

**Fig. 1:**
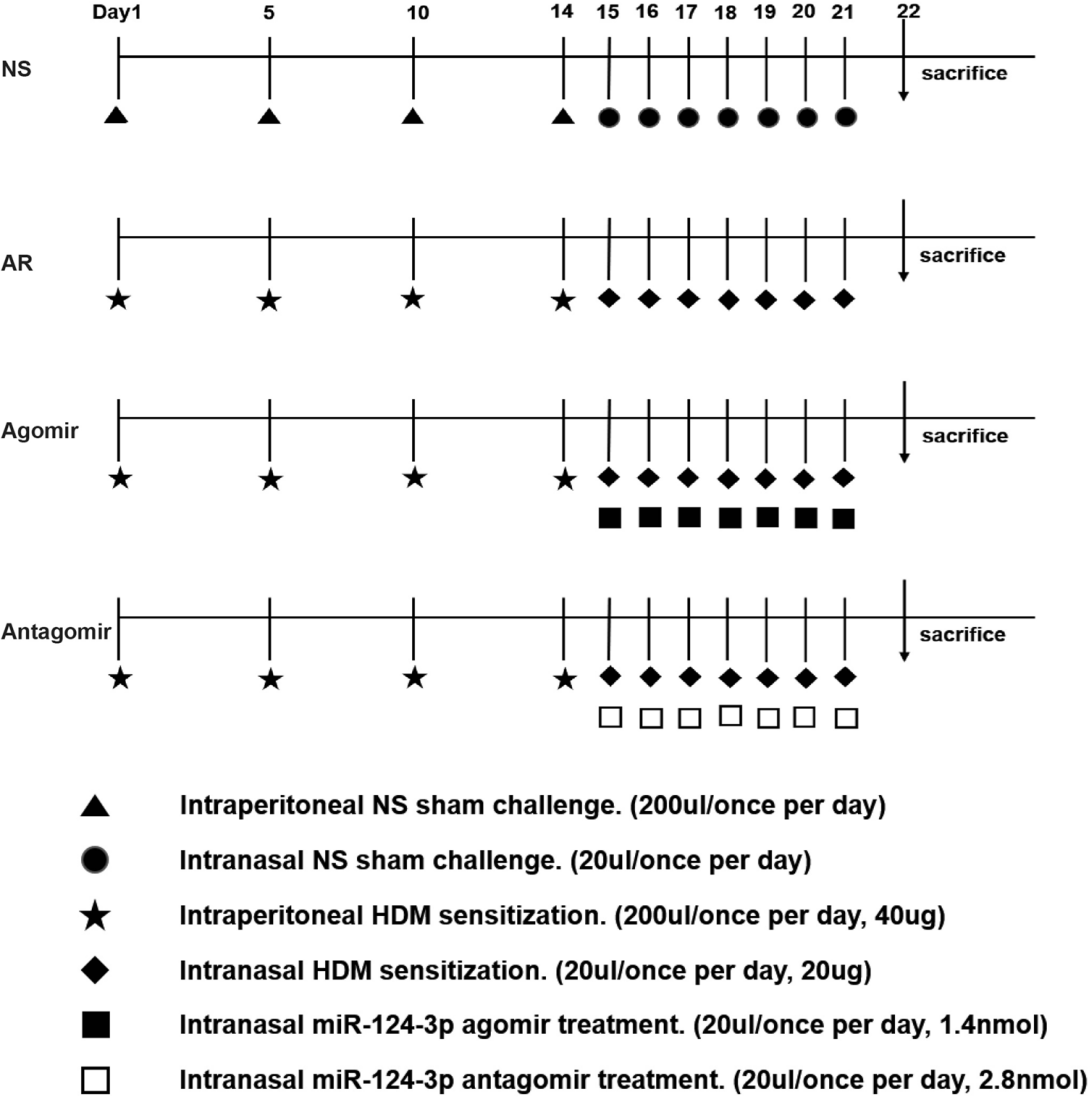
Schematic for the establishment of AR mice and treatment schedule

The flowchart of mouse allocation and treatments is shown in Fig. 1. Briefly, the mice were sensitized using house–dust–mite (HDM) antigen as follows: 40 µg of HDM extract (D. pteronyssinus, Greer Labs) diluted in 200 µL of sterile normal saline was administered to the mice by four intraperitoneal injections on days 1, 5, 10 and 14. Intranasal challenge was performed using 20 µg of HDM diluted in 20 µL of normal saline (NS) once a day from days 15 to 21. The allergic symptom score was calculated 15 min after the last challenge on day 21.

In detail, 24 mice were randomly allocated into four groups: NS (*n* = 6), AR (*n* = 6), agomir (*n* = 6), and antagomir (*n* = 6). The mice in the NS group were sensitized and intranasally challenged with NS only, and those in the AR group were sensitized by intraperitoneal injections and intranasally challenged with HDM allergen. The sensitized mice in the agomir group and antagomir groups received the intranasal HDM challenge and then a nasal administration of 20 µL of 1.4 nmol miR-124-3p agomir or 20 µL of 2.8 nmol antagomir (GenePharma, Shanghai) once a day from days 15 to 21.

The mice were euthanized 24 h after the final challenge, peripheral blood was obtained by extracting the eyeball, and the nasal mucosa and spleen were isolated by precise dissection.

### Measurement of HDM-specific IgE

Serum from mice was prepared by centrifugation of blood and then cryopreserved at − 80 °C. The serum level of HDM-specific IgE was measured using an enzyme-linked immunosorbent assay (ELISA) kit (Chondrex, Redmond, WA, USA) according to the manufacturers’ protocol.

### Histopathology

Fresh murine nasal mucosa samples were fixed with 4% paraformaldehyde overnight, decalcified in EDTA, embedded in paraffin, dewaxed, rehydrated, and used for hematoxylin and eosin (H&E) staining, immunohistochemistry and immunofluorescence analyses. The staining was performed using standard procedures.

### Immunohistochemistry (IHC)

The nasal mucosa tissue sections were blocked in blocking buffer for 1 h and then incubated with goat anti-rabbit IL-4 overnight at 4 °C. The sections were washed with PBS supplemented with 0.1% Tween-20 (PBS-T) and incubated with HRP-conjugated donkey anti-goat IgG antibody for 1 h at room temperature. After staining, all the samples were washed with PBS-T and then mounted for imaging. Images were captured using a microscope (Olympus, Japan).

### Immunofluorescence (IF) staining

Freshly dissected murine nasal mucosa specimens were collected and processed as described above. Sections were deparaffinized, subjected to antigen repair, blocked with BSA blocking buffer for 30 min and then stained with rabbit anti-IL-4Rα (Thermo Fisher) overnight. Donkey–anti-rabbit FITC (Proteintech, China) was used as the secondary antibody. DAPI (Proteintech, China) was used for counterstaining. The slides were sealed with anti-fluorescence quencher. All images were captured with a confocal microscope (Leica, Germany).

### Isolation of splenic lymphocytes

Splenic lymphocytes were isolated by Ficoll–Hypaque density centrifugation. Briefly, the spleens were mechanically minced into a homogenous paste with a scalpel on a dish plate and washed with PBS containing 2% FBS. After incubation in a 24-well plate for 30 min at 37 °C in a humidified incubator, cell suspensions were passed through a 70 µm Falcon nylon cell strainer, and lymphocytes were isolated from single cell suspensions of the spleen using lymphocyte separation medium (TBD, China). The isolated lymphocytes were used for further experiments, such as flow cytometry.

### In vitro culture of lymphocytes and treatment protocol

Splenic lymphocytes from mice or human PBMCs were cultured in RPMI 1640 medium supplemented with 10% FBS, 1 µg/mL anti-CD3 (BioLegend, USA), 1 µg/mL anti-CD28 (BioLegend, USA) and 100 U/mL mouse IL-2 (PeproTech, USA) in a humidified incubator containing 5% CO_2_ at 37 °C. They were transferred to a 24-well plate at 1 × 10^6^ cells/well in medium for 16 h and then transiently transfected with 2.5 µg of miR-124-3p mimic or mimic negative control or 5 µg of inhibitor or inhibitor negative control using Lipofectamine 3000 according to the manufacturer’s protocol for suspension cells. Each transfection was performed in triplicate in 24-well plates. Twenty-three hours after transfection, 2.5 µg of HDM antigen was added to the medium and incubated for 1 h. The cells in each well were then collected for extraction of RNA and protein.

### Real-time PCR

Total RNA from murine nasal mucosa, splenic lymphocytes and human PBMCs was extracted using TRIzol reagent (Invitrogen, USA). RNA was reverse transcribed using ReverTra Ace^®^ qPCR RT Master Mix with gDNA Remover (Toyobo, Japan). Real-time PCR was performed using KOD SYBR qPCR Mix (Toyobo, Japan), and detection was achieved using the LightCycler 480 System (Roche, Switzerland) according to the manufacturer’s instructions. All data were normalized to the β-actin levels and expressed relative to the control, and relative expression was calculated using the equation 2^−ΔΔCt^. The primer sequences are presented in Supplementary Table 1.

### Western blot

Protein lysates from splenic lymphocytes and human PBMCs were processed in RIPA buffer (Kaiji Biotech, China) in the presence of phosphatase inhibitor and protease inhibitor cocktails. The total protein concentrations were measured by BCA assay (Kaiji Biotech, China). The samples were separated on 8–12% SDS–PAGE gels and then transferred to PVDF membranes (Millipore, USA). The membrane was incubated with primary antibody overnight at 4 °C and then with IRDye secondary antibodies (LI-COR Biosciences, USA) for 1 h at room temperature. The membrane was subsequently subjected to three 10-min washes with TBST buffer. The bands were scanned and quantified using a LI-COR Odyssey CLx scanner (LI-COR Biosciences, USA). β-Actin or Gapdh was used as an internal reference.

### Flow cytometry

Flow cytometric analyses for the T cell phenotyping of splenic lymphocytes were performed as follows. Prior to cell surface staining, cells (1 × 10^6^) were seeded into a 24-well plate and stimulated with PMA/Ionomycin mixture and BFA/monensin mixture for 4 h. For surface marker staining, cells (1 × 10^6^) were stained with anti-CD4 conjugated with APC-Cyanine7 (eBioscience, USA) for 30 min on ice and then washed in PBS containing 5% FBS. For intracellular cytokine staining, cells (1 × 10^6^) were fixed with the FoxP3/Transcription Factor Staining Buffer Kit (eBioscience, USA) and then incubated with anti-IL4 conjugated with PE-Cyanine7 and anti-IFN-γ conjugated with PerCP-Cyanine5.5 (eBioscience, USA) for 30 min. Cytokine level in CD4+ lymphocytes was analyzed using FlowJoV10 (Tree Star, Ashland, OR).

### Dual-luciferase reporter assays

**Fig. 2:**
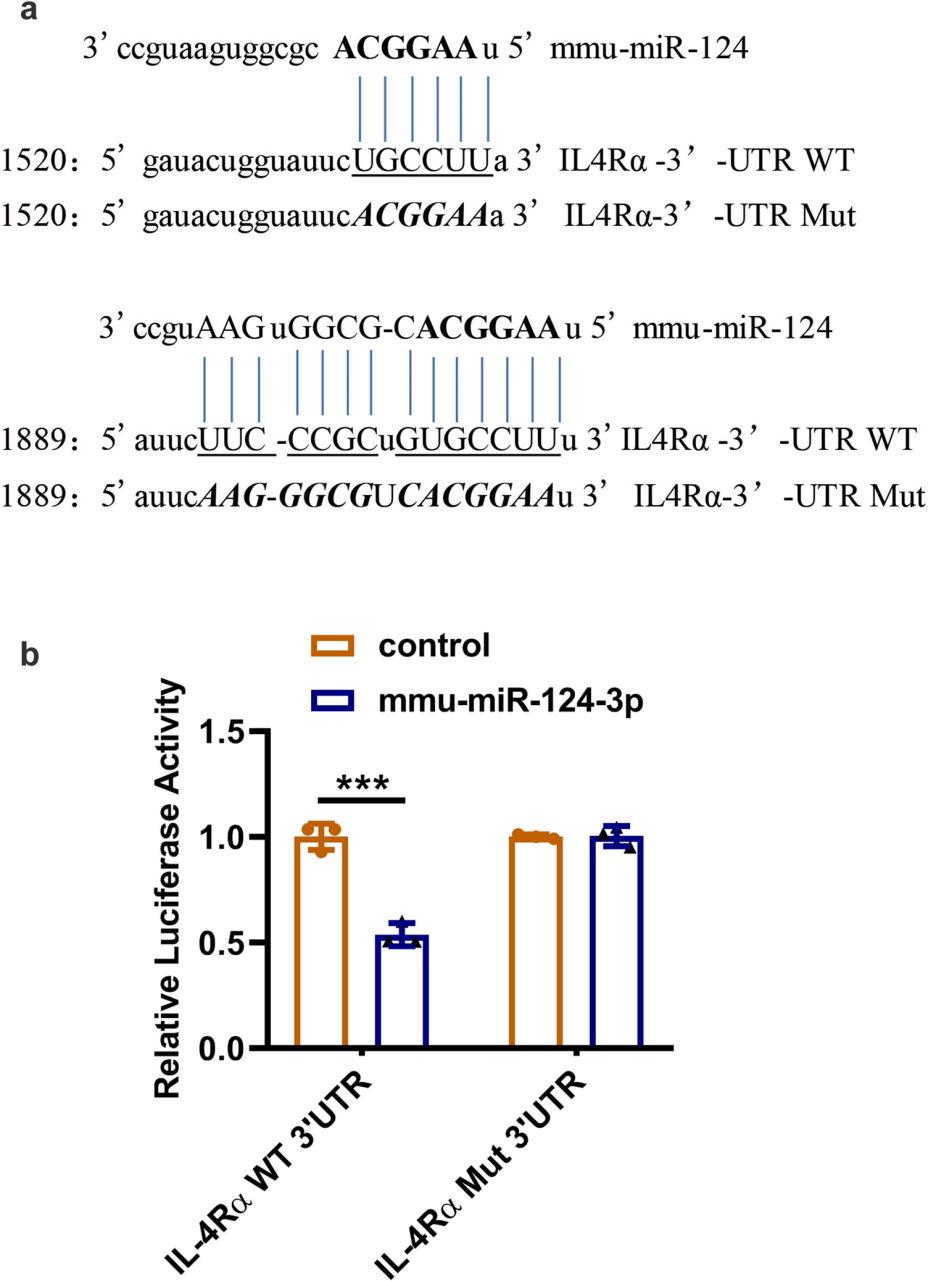
miR-124-3p directly interacts with IL-4Rα by binding to its 3′-UTR. **a** Predicted binding sequences and mutated sequences between miR-124-3p and IL-4Rα. **b** Relative luciferase activity after cotransfection of mmu-miR-124-3p mimics/controls and the IL-4Rα wild/mutant type (WT/Mut) reporter vector (*n* = 3). ***p < 0.001

IL-4Rα was predicted to be the underlying target of miR-124p by an online bioinformatics analysis (PicTar, TargetScan and miRBase). An interaction diagram of mmu-miR-124 and wild-type IL-4Rα-UTR is shown in Fig. 2a. Dual-luciferase assays were implemented using the luciferase reporter assay system in accordance with the manufacturer’s instructions (Promega). The mutated (Mut) or wild-type (WT) IL-4Rα-3′-UTR sequence including the mmu-miR-124 targeting site was inserted into the XhoI/BamHI sites of the pLUC vector to construct pLUC-luc-IL-4Rα. All constructs were verified by sequence analysis (Supplementary Fig. 1). HEK-293T cells were prepared, seeded in 96-well plates and then transfected with pLUC-luc-IL-4Rα (0.2 µg), mmu-miR-124 negative or mimic control (0.45 µg) or Renilla luciferase (0.15 µg) by adopting FuGENE^®^ HD (Roche, Switzerland). The transfections were performed in duplicate, and each experiment was repeated in triplicate. Luciferase activity was detected after 48 h using the Luciferase Reporter Assay System (Promega). IL-4Rα-3′-UTR activity is expressed as a ratio of firefly luciferase activity to Renilla luciferase activity.

### Statistical analyses

All quantification results are shown as the means (± SEMs) of at least three independent experiments. Statistical comparisons between two groups were conducted by unpaired two-tailed Student’s *t* tests. One-way analysis of variance (ANOVA) was used for comparisons of multiple groups. The statistical analyses were performed using GraphPad Prism 8 software (GraphPad Software Inc.). A *p* value < 0.05 was considered to indicate statistical significance.

## Results

### miR-124-3p directly targets IL-4Rα

Based on a previous bioinformatics analysis, IL-4Rα was identified as a novel target of miR-124-3p. To evaluate whether miR-124-3p directly targets IL-4Rα mRNA, we first searched the 3’-UTR sequence of IL-4Rα as the potential binding site of miR-124-3p (Fig. 2a). We then performed a luciferase reporter assay to predict the interaction between miR-124-3p and IL-4Rα (Fig. 2b). We found that miR-124-3p significantly reduced the luciferase activity in the IL-4Rα WT group, but no change in luciferase activity was detected in the IL-4Rα Mut group with mutation in the predicted miR-124-3p targeting sequence in the reporter vector (pLUC-IL4RαMut-3′-UTR). This result indicates that miR-124-3p may directly target IL-4Rα by binding to 3′-UTR sites.

### Increased miR-124-3p lowered the levels of IL-4Rα and type 2 cytokines in lymphocytes in vitro

**Fig. 3:**
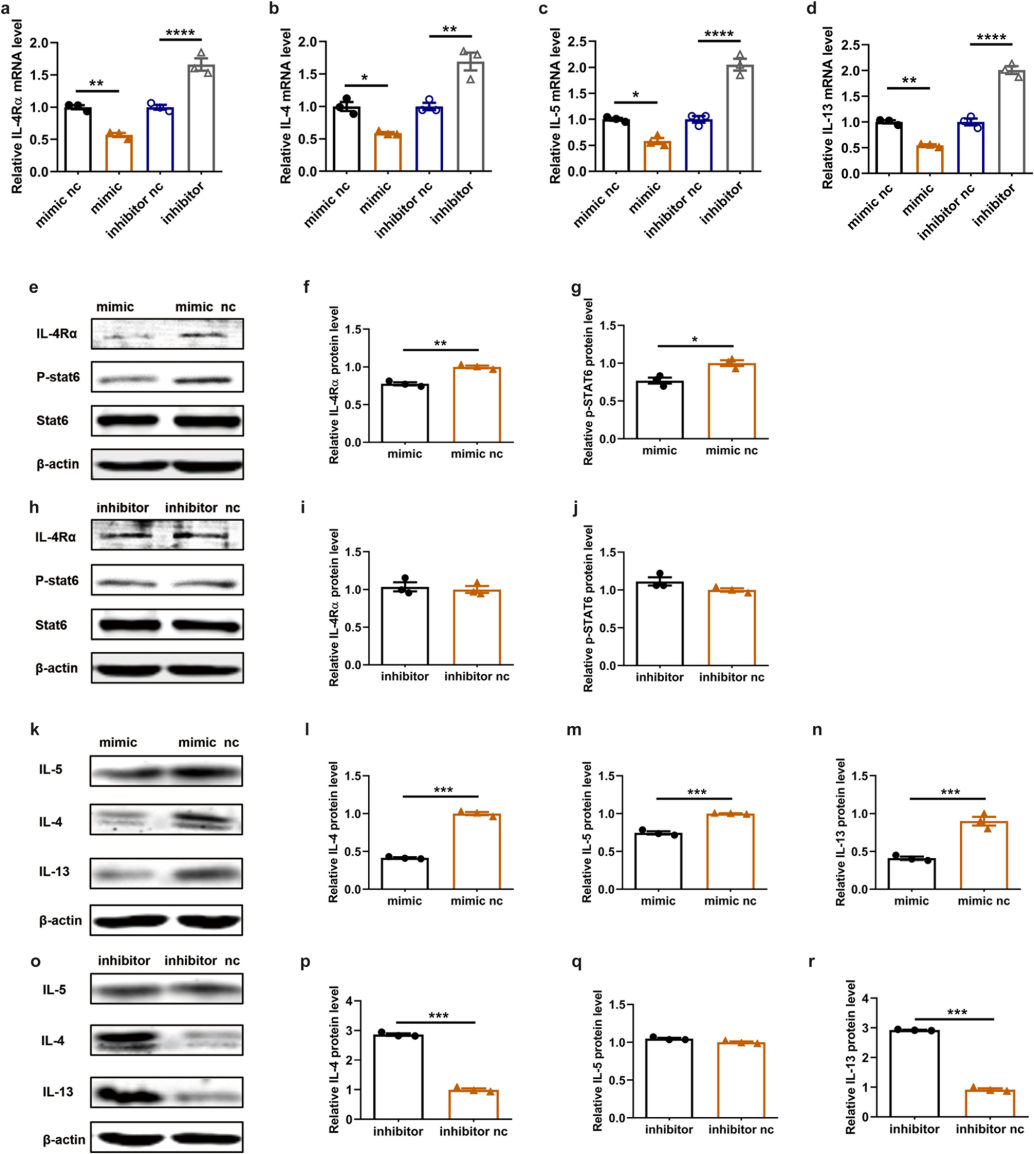
miR-124-3p decreases the mRNA and protein expression levels of IL-4Rα and type 2 cytokines in vitro (*n* = 3). The effect of miR-124-3p mimic and inhibitor on mRNA expression of **a** IL-4Rα, **b** IL-4, **c** IL-5 and **d** IL-13 splenic lymphocytes determined by quantitative RT-PCR. **e**–**g** Effect of miR-124-3p mimic on protein expression of IL-4Rα and p-STAT6. **e** Representative images and histogram of **f** IL-4Rα and **g** p-STAT6 after treatment with miR-124-3p mimic. **h**–**j** Effect of miR-124-3p inhibitor on protein expression of IL-4Rα and p-STAT6. **h** Representative images and histogram of **i** IL-4Rα and **j** p-STAT6 after treatment with miR-124-3p mimic. **k**–**n** Effect of miR-124-3p mimic on protein expression of IL-4, IL-5 and IL-13. **k** Representative images and histogram of **l** IL-4, **m** IL-5 and **n** IL-13 after treatment with miR-124-3p mimic. **o**–**r** Effect of miR-124-3p inhibitor on protein expression of IL-4, IL-5 and IL-13. **o** Representative images and histogram of **p** IL-4, **q** IL-5 and **r** IL-13 after treatment with miR-124-3p mimic. *p < 0.05, **p < 0.01 and ***p < 0.001

To investigate whether miR-124-3p directly downregulates IL-4Rα and further reduces the levels of type 2 cytokines in vitro, splenic lymphocytes from mice were cultured and transfected with miR-124-3p mimic and miR-124-3p inhibitor. First, we confirmed that the miR-124-3p mimic decreased both the mRNA and protein expression of IL-4Rα in splenic lymphocytes (Fig. 3a, e, f). Similarly, we found that the transcription factor p-STAT6, which is crucial for the induction of type 2 immune responses, was decreased after miR-124-3p mimic treatment (Fig. 3e, g). Furthermore, both the mRNA and protein levels of type 2 cytokines (IL-4, IL-5 and IL-13) were decreased in splenic lymphocytes after treatment with the miR-124-3p mimic (Fig. 3b–d, k–n). In contrast, the miR-124-3p inhibitor significantly increased the mRNA level of both IL-4Rα and type 2 cytokines (Fig. 3a–d). However, the protein levels of IL-4Rα, p-STAT6 and IL-5 were increased, albeit not significantly, after miR-124-3p inhibitor treatment (Fig. 3h–j, o, q). Interestingly, the protein levels of IL-4 and IL-13 were further increased after miR-124-3p inhibitor treatment (Fig. 3o, p, r).

### Increased miR-124-3p attenuated type 2 inflammatory response in human PBMCs in vitro

**Fig. 4:**
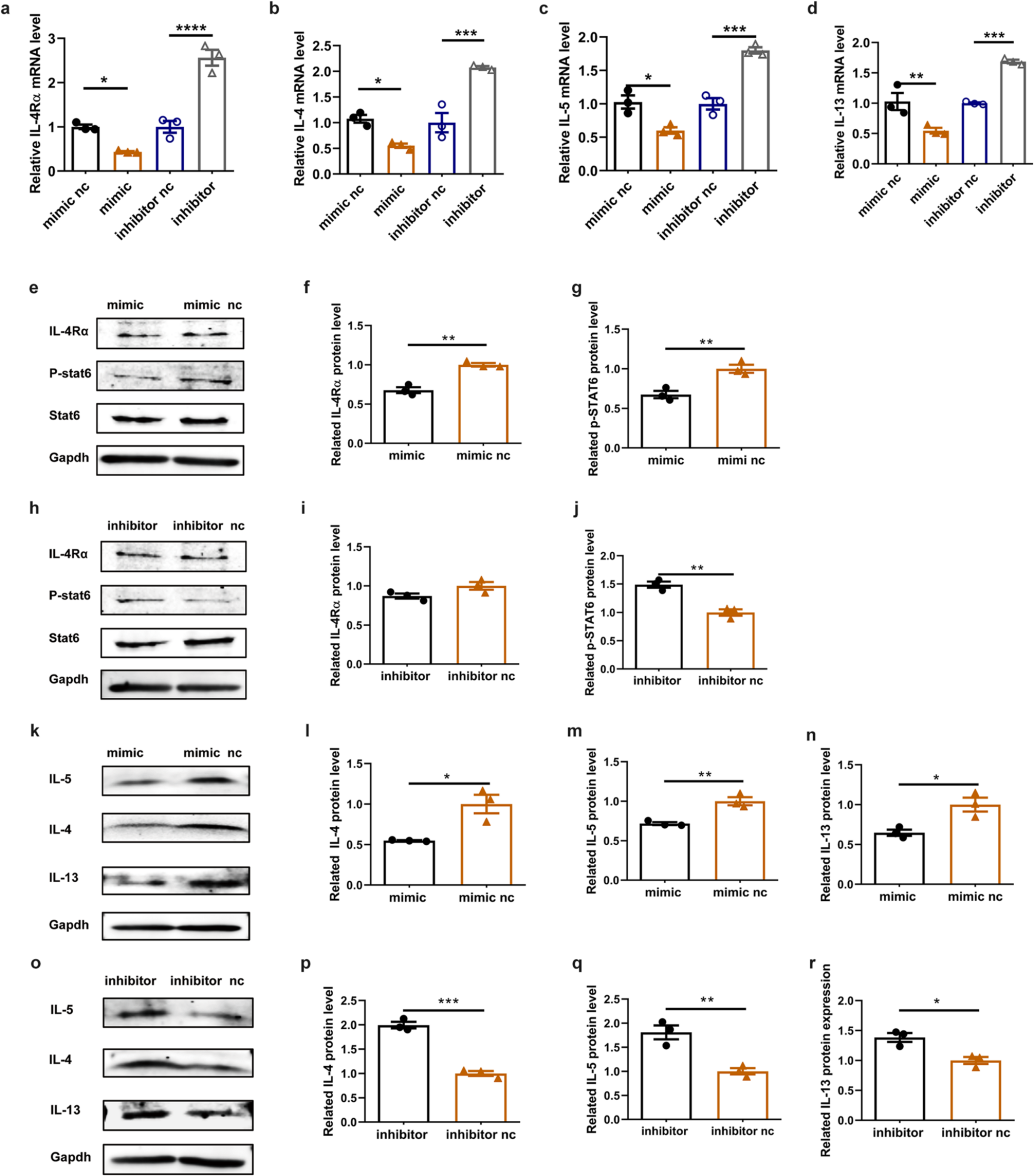
miR-124-3p reduces the mRNA and protein expression of IL-4Rα and type 2 cytokines in human PBMCs (*n* = 3). The effect of miR-124-3p mimic and inhibitor on mRNA expression of **a** IL-4Rα, **b** IL-4, **c** IL-5 and **d** IL-13 in lymphocytes determined by quantitative RT-PCR. **e**–**g** Effect of miR-124-3p mimic on protein expression of IL-4Rα and p-STAT6. **e** Representative images and histogram of **f** IL-4Rα and **g** p-STAT6 after treatment with miR-124-3p mimic. **h**–**j** Effect of miR-124-3p inhibitor on protein expression of IL-4Rα and p-STAT6. **h** Representative images and histogram of **i** IL-4Rα and **j** p-STAT6 after treatment with miR-124-3p mimic. **k**–**n** Effect of miR-124-3p mimic on protein expression of IL-4, IL-5 and IL-13. **k** Representative images and histogram of **l** IL-4, **m** IL-5 and **n** IL-13after treatment with miR-124-3p mimic. **o**–**r** Effect of miR-124-3p inhibitor on protein expression of IL-4, IL-5 and IL-13. **o** Representative images and histogram of **p** IL-4, **q** IL-5 and **r** IL-13 after treatment with miR-124-3p mimic. *p < 0.05, **p < 0.01 and ***p < 0.001

To more comprehensively validate the role of miR-124-3p in the type 2 inflammatory response, we also transfected human PBMCs with miR-124-3p mimic and miR-124-3p inhibitor. The results were similar to those found in mouse splenic lymphocytes. The miR-124-3p mimic decreased both the mRNA and protein expression levels of IL-4Rα and protein level of p-STAT6 in human PBMCs (Fig. 4a, e, f, g). Furthermore, both the mRNA and protein levels of type 2 cytokines (IL-4, IL-5 and IL-13) were decreased in human PBMCs after treatment with the miR-124-3p mimic (Fig. 4b–d, k–n). In contrast to the effect of the mimic, the miR-124-3p inhibitor markedly elevated the mRNA levels of both IL-4Rα and type 2 cytokines (Fig. 4a–d, o–r). However, the protein level of IL-4Rα was unchanged after miR-124-3p inhibitor treatment (Fig. 4h, i). However, the protein levels of p-STAT6 and type 2 cytokines were further increased after miR-124-3p inhibitor treatment (Fig. 4h, j, o–r).

### Increased miR-124-3p lowered the IL-4Rα and IL-4 cytokine levels in vivo

**Fig. 5:**
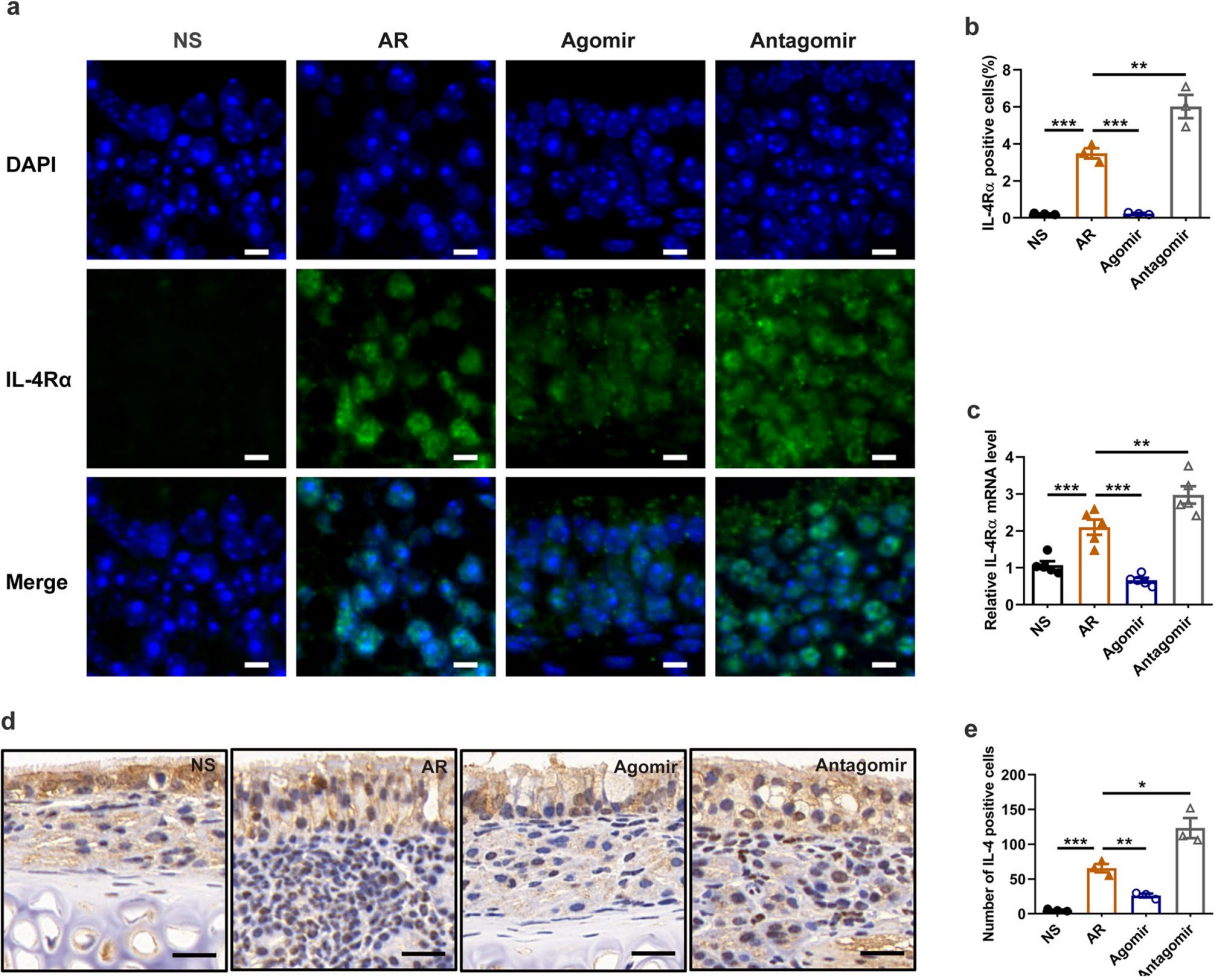
miR-124-3p decreases IL-4Rα and IL-4 cytokine expression in the nasal mucosa. **a** Representative images of IL-4Rα (green) expression in the nasal mucosa obtained by immunofluorescence labeling (×400). DAPI (blue), represents nuclear staining. The scale bar represents 10 μm. **b** Histogram of the percentage of IL-4Rα positive cells in nasal mucosa tissue sections detected by immunofluorescence staining (*n* = 3). **c** IL-4Rα mRNA expression in the nasal mucosa detected by quantitative RT-PCR (*n* = 5). **d** Representative images of IL-4 expression in the nasal mucosa obtained by immunohistochemical staining (×400). The scale bar represents 20 μm. **e** Histogram of the number of IL-4 positive cells in nasal mucosa tissue sections detected by immunohistochemistry (*n* = 3). *p < 0.05, **p < 0.01 and ***p < 0.001

To further validate the function of miR-124-3p in vivo, we established the HDM-induced AR mouse model and treated the mice with the miR-124-3p agomir and antagomir. The detailed protocol is shown in Fig. 1. The expression of IL-4Rα in the nasal mucosa was determined by immunofluorescence. As shown in Fig. 5a, b, compared with the control group, the percentage of IL-4Rα positive cells in the nasal mucosa was significantly increased in the AR group. Treatment with the miR-124-3p agomir and antagomir markedly reduced and elevated the expression of IL-4Rα in the nasal mucosa of AR mice, respectively. Furthermore, the IL-4Rα mRNA level in the nasal mucosa of AR mice was decreased and increased after miR-124-3p agomir and miR-124-3p antagomir treatment, respectively (Fig. 5c). In addition, we examined the expression of IL-4 in the nasal mucosa by immunohistochemistry (Fig. 5d). Similarly, the number of IL-4 positive cells in the nasal mucosa of AR mice was significantly decreased and increased by miR-124-3p agomir and miR-124-3p antagomir treatment, respectively (Fig. 5e).

### Increased miR-124-3p alleviated Th2 cell differentiation of splenic lymphocytes in vivo

**Fig. 6:**
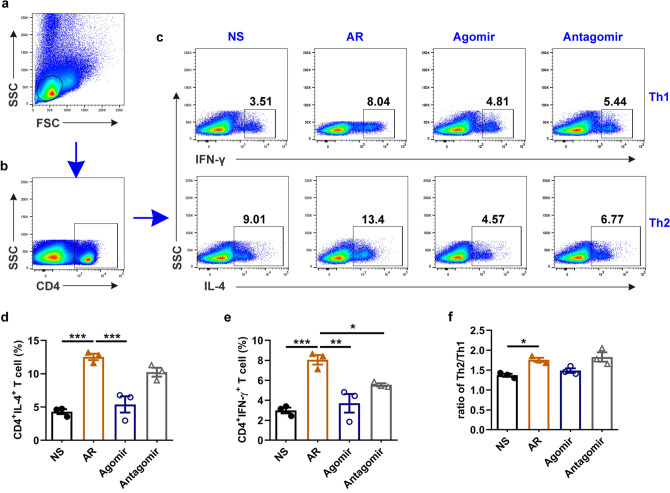
miR-124-3p alleviates Th2 cell differentiation of splenic lymphocytes in vivo (*n* = 3). **a**–**c** Gating strategy for Th1 and Th2 cells. **a** Gating for lymphocytes. **b** Gate for CD4^+^ T cells. **c** Representative dot plots of the percentages of Th1 and Th2 cells. **d** Percentage of Th2 cells. **e** Percentage of Th1 cells. **f** Ratio of Th2 to Th1 subsets. *p < 0.05, **p < 0.01 and ***p < 0.001

To verify whether miR-124-3p affects Th2 cell differentiation in vivo, we examined the proportions of Th1/Th2/Th17/Tregs in the splenocytes of mice by flow cytometry. The results showed high increases in the Th2 and Th1 percentages and Th2/Th1 ratio in the AR group compared with the control group, which indicated the development of a Th2 inflammatory response in HDM-sensitized AR mice (Fig. 6). Subsequently, the Th2 inflammatory response was attenuated in AR mice treated with the miR-124-3p agomir (Fig. 6c, d). In addition, the Th2 percentage and Th2/Th1 ratio of the antagomir group were higher than those of the agomir group, but the difference was not significant (Fig. 6d, f). On the other hand, the proportions of Th17 cells and Tregs were not significantly different among these groups (Supplementary Fig. 2).

### Increased miR-124-3p attenuated the nasal allergic response

**Fig. 7:**
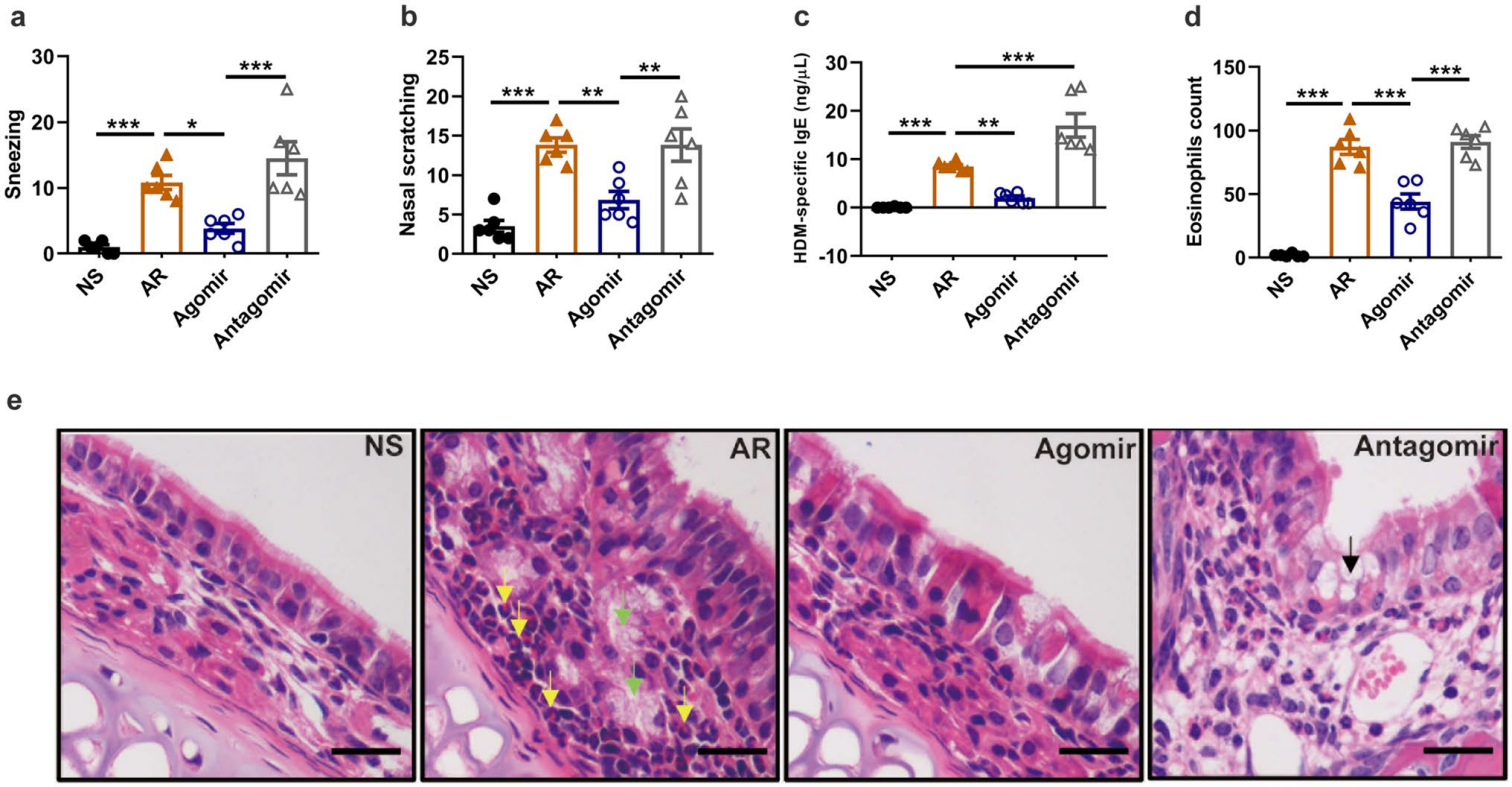
miR-124-3p protects against HDM-induced allergic response in allergic rhinitis mice. **A**, **B** Number of (*a*) sneezing and (*b*) nasal scratching after treatment with miR-124-3p agomir/antagomir. (*c*) HDM-specific IgE levels in serum. (*d*) Eosinophil counts detected in H&E-stained nasal mucosa tissue sections (200× magnification). (*e*) Representative H&E staining of the nasal mucosa (400×). The yellow arrows indicate eosinophils, the green arrows indicate mucus glands, and the black arrows indicate goblet cells. The scale bar represents 20 μm. HDM, house dust mite. NS, control group challenged with normal saline (*n* = 6). AR, allergic rhinitis group challenged with HDM (*n* = 6). Agomir, AR group treated with miR-124-3p agomir (*n* = 6). Antagomir, AR group treated with miR-124-3p antagomir (*n* = 6). Each dot represents one mouse. *p < 0.05, **p < 0.01 and ***p < 0.001

To evaluate the effect of miR-124-3p on the nasal allergic response in AR mice, we examined the nasal symptoms, serum HDM-specific IgE levels, and eosinophil infiltration in the nasal mucosa. As shown in Fig. 7a, b, the numbers of sneezing and nasal scratching were markedly higher in the AR group than in the control group but were decreased significantly in the miR-124-3p agomir group compared with the AR group. The antagomir group exhibited a higher number of sneezing and nasal scratching than the agomir group, but the differences were not significant compared the numbers found for the AR group. Furthermore, the high level of serum HDM-specific IgE in the AR group was significantly decreased and increased after treatment with the miR-124-3p agomir and antagomir, respectively (Fig. 7c). Moreover, the number of infiltrated eosinophils in the lamina propria of the nasal mucosa was observed by H&E staining. As shown in Fig. 7d, e, the eosinophil count in the lamina propria of AR group was higher than that of the control group. Compared with the AR group, the eosinophil count was significantly decreased in the agomir group but not changed significantly in the antagomir group; however, the antagomir group had higher eosinophil counts than the agomir group.

## Discussion

AR is a global disease with increasing prevalence that places a great burden on the quality of life of patients [1–3]. Type 2 inflammation was recently reported as the main pathophysiological mechanism of AR [11]. Finding a therapeutic target for reducing type 2 inflammation is important for AR treatment. Herein, we found that miR-124-3p may be a potential target in AR treatment. Increased miR-124-3p downregulates IL-4Rα expression and further attenuates type 2 inflammation in AR mice, as demonstrated by decreases in the IL-4, IL-5, and IL-13 cytokine levels, Th2 differentiation, and p-STAT6 expression in nasal mucosa and lymphocytes.

It has been reported that miRNAs play a critical role in allergic diseases, including asthma and AR [8]. Our previous study screened some differential genes in the nasal mucosa and found that the miR-124-3p levels were decreased in AR mice and increased in anti-inflammatory mice treated with ipratropium bromide [9]. To investigate whether miR-124-3p might be a therapeutic target in AR, we first predicted the possible functional genes that bind to miR-124-3p via bioinformatic analysis. The results showed that IL-4Rα is a putative target gene of miR-124-3p. The dual-luciferase reporter assay further confirmed that miR-124-3p directly binds to the 3’UTR of IL-4Rα. These results indicated that miR-124-3p might downregulate AR via IL-4Rα signaling, which plays a role in promoting type 2 inflammation. MiR-124 is reportedly expressed in various tissues and is particularly highly expressed in immune cells and organs, including peripheral blood mononuclear cells, bone marrow, lymph nodes, and thymus [12–15]. A previous study showed that miR-124 could suppress CD4^+^ T cell immunoactivity by targeting interferon regulatory factor 1 (IRF1) [16]. Moreover, miR-124 exerts a broad antiproliferative effect, and robustly inhibits transactivation of nuclear factor of activated T cells (NFAT) activity [17]. Thus, miR-124 expression may function as a negative regulator to control inflammation. Similar to our previous study, miR-124-3p is reportedly decreased in the nasal mucosa of AR mice and patients with AR [10]. Furthermore, recent studies showed that the overexpression of miR-124 could dramatically inhibit the activation of NF-kB and the expression of inflammatory factors to regulate innate immunity [18, 19]. However, the effect of miR-124-3p on immune response, particularly type 2 inflammation, in AR is unclear.

To clarify the downregulation of miR-124-3p in type 2 inflammation in AR, we examined the expression of IL-4Rα and type 2 cytokines (IL-4, IL-5 and IL-13) in vitro. The expression of IL-4Rα, p-STAT6, IL-4, IL-5 and IL-13 was significantly reduced in splenic lymphocytes after miR-124-3p mimic treatment, and the miR-124-3p inhibitor rescued these changes. IL-4Rα, a common receptor shared by IL-4 and IL-13, signals via STAT6 and plays an important role in type 2 allergic immunity [20–22]. In addition, in this study, we isolated human PBMCs from healthy individuals for in vitro culture exposed to miR-124-3p. Similar to the results found for mouse splenocytes, miR-124-3p also suppressed the type 2 inflammatory response in human PBMCs. Therefore, the inhibitory effects of miR-124-3p on type 2 inflammation in AR may be mediated by the IL-4Rα/STAT6 signaling pathway.

To verify the suppressive immune regulation of miR-124-3p in AR, HDM-induced AR mice were established. We found that the miR-124-3p agomir reduced IL-4Rα and IL-4 expression in the nasal mucosa, Th2 differentiation in splenic lymphocytes and the allergic response in AR mice. Furthermore, the miR-124-3p antagomir significantly increased the IL-4Rα and IL-4 levels and aggravated the allergic response, including nasal symptoms, serum sIgE levels and eosinophil infiltration in the nasal mucosa. These in vivo experiments demonstrated that miR-124-3p may alleviate type 2 inflammation and further attenuate the allergic response in AR. miR-124-3p plays a critical role in anti-inflammation and can reduce the production of proinflammatory cytokines, including IL-6 and TNF-α [23–25]. Similarly, a study on asthma indicated that miR-124 may be involved in eosinophilic inflammation and has good diagnostic value for asthma [26]. Moreover, it has been reported that the upregulation of miR-124-3p improves eosinophil infiltration, inhibits apoptosis of the nasal mucosa in AR mice and alleviates allergic nasal symptoms [10]. However, there is no report on the regulation of type 2 inflammation by miR-124-3p in AR. We investigated the immune regulation mediated by miR-124-3p in AR mice and the possible mechanism involved using both in vitro and in vivo methods, and the results will provide novel therapeutic evidence for AR.

In conclusion, miR-124-3p might attenuate type 2 inflammation in AR by downregulating IL-4Rα signaling. Our findings may be beneficial for advancing the clinical application of miRNAs in AR treatment, and miR-124-3p may be a novel therapeutic target in AR.

## Supplementary Information

Below is the link to the electronic supplementary material.

Supplementary file1 (DOC 1874 KB)
