# Supplementary material for: Increased miR-124-3p alleviates type 2 inflammatory response in allergic rhinitis via IL-4Rα
Source: Inflamm Res. 2022 Aug 3;71(10-11):1271–82. doi: 10.1007/s00011-022-01614-x (PMC9616750; doi:10.1007/s00011-022-01614-x)

Table 1. Information of primer sequences.

| Genes | | Sequence(5’ to 3’) |
| --- | --- | --- |
| Mouse IL-4 | Forward | TACCAGGAGCCATATCCACGGATG |
|  | Reverse | TGTGGTGTTCTTCGTTGCTGTGAG |
| Mouse IL-4Rα | Forward | GCTTTGGTATTGTGTACTCGTC |
|  | Reverse | GGATGGTGATCTGTCATCGTAG |
| Mouse IL-5 | Forward | CTCTGTTGACAAGCAATGAGAC |
|  | Reverse | GTCTAGCCCCTGAAAGATTTCT |
| Mouse IL-13 | Forward | ACCCTTAAGGAGCTTATTGAGG |
|  | Reverse | ATTGCAATTGGAGATGTTGGTC |
| Mouse β-actin | Forward | CTACCTCATGAAGATCCTGACC |
|  | Reverse | CACAGCTTCTCTTTGATGTCAC |
| Human IL-4 | Forward | AAAACTTTGAACAGCCTCACAG |
|  | Reverse | GGTTTCCTTCTCAGTTGTGTTC |
| Human IL-4Rα | Forward | AAGCCCAGCGAGCATGTGAAAC |
|  | Reverse | TCCAGGTCAGCAGCAGAGTGTC |
| Human IL-5 | Forward | ACTCATCGAACTCTGCTGATAG |
|  | Reverse | TGTGCCTATTCCCTGAAAGATT |
| Human IL-13 | Forward | CATGTCCGAGACACCAAAATC |
|  | Reverse | CCCTCGCGAAAAAGTTTCTTTA |
| Human β-actin | Forward | GCACTCTTCCAGCCTTCCTTCC |
|  | Reverse | GCGGATGTCCACGTCACACTTC |

Fig.1 Sequence analysis to verify the constructs.


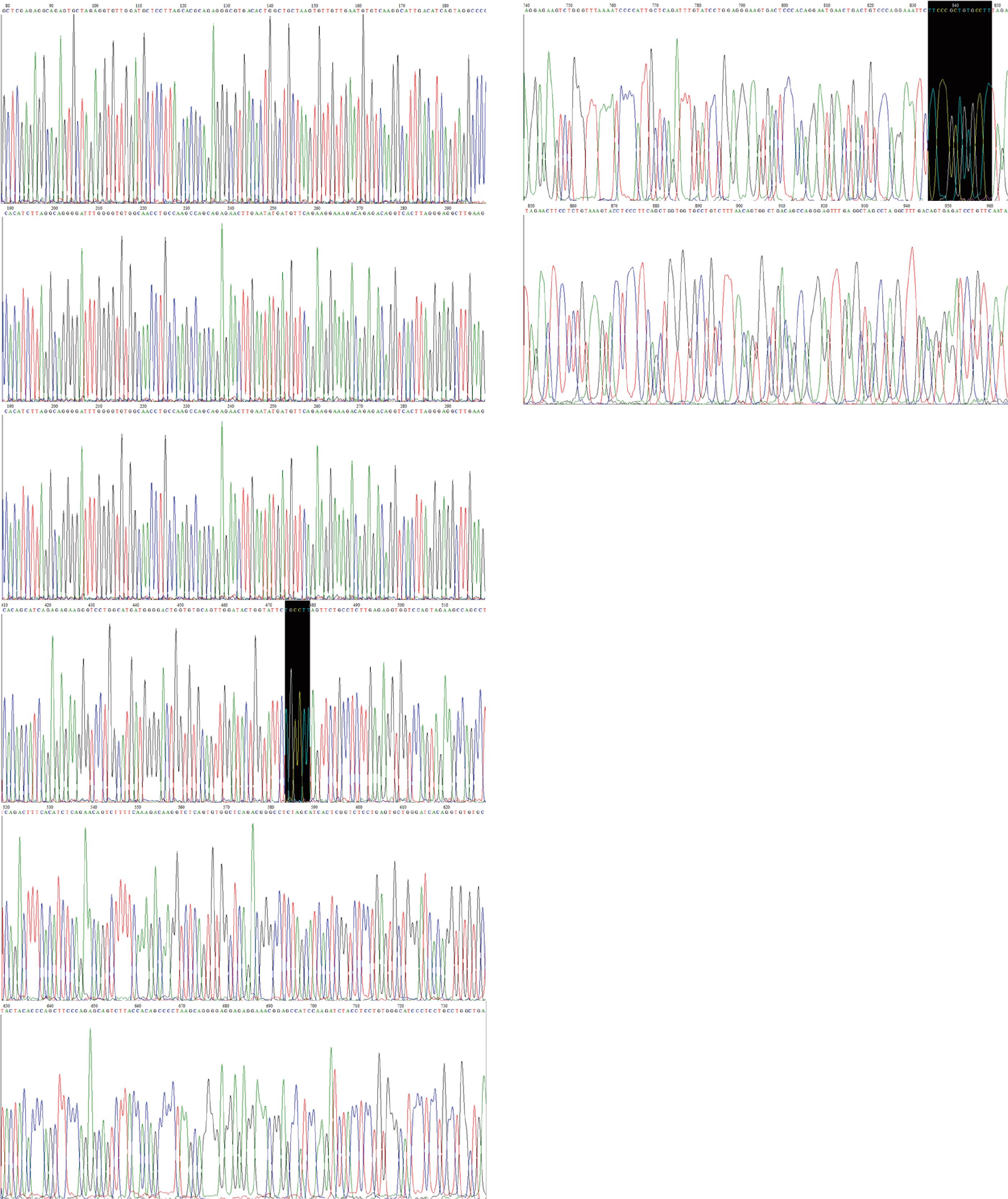


Fig.2 Percentages of Th17 and Treg cells in CD4^+^ T cells.


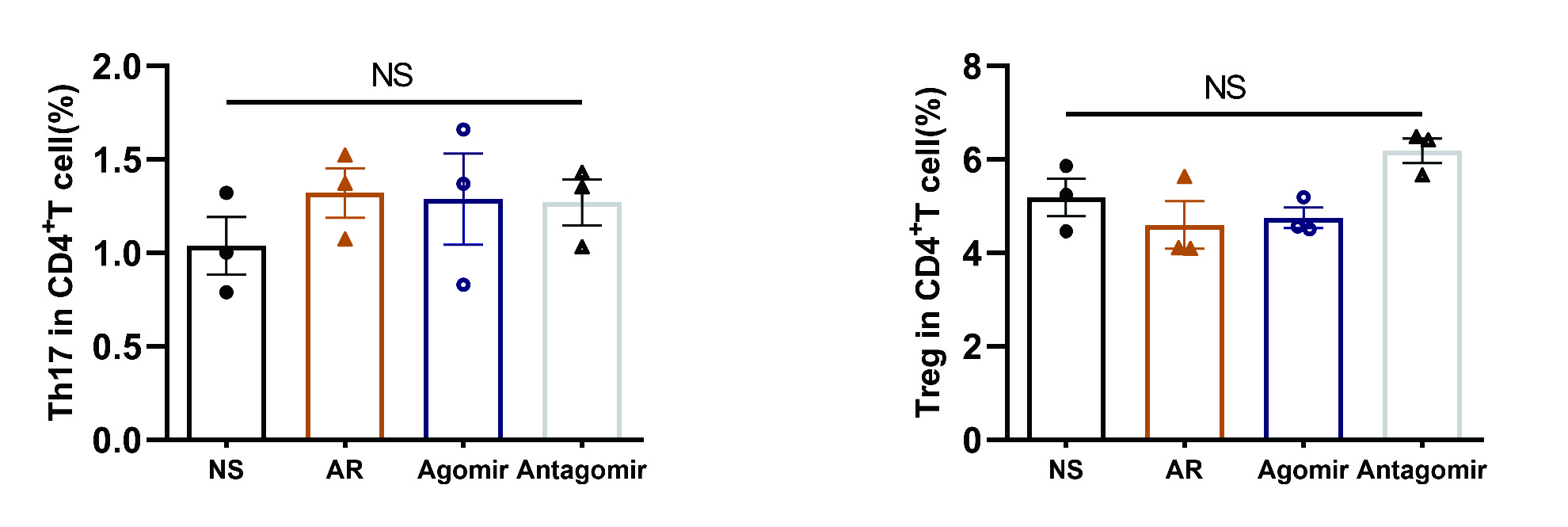

Supplement: Supplementary file 1 — Supplementary file1 (DOC 1874 KB) [file 11_2022_1614_MOESM1_ESM.doc]
